# Spontaneous Lung Herniation Leading to Extensive Subcutaneous Emphysema, Pneumothorax, Pneumomediastinum, and Pneumopericardium

**DOI:** 10.7759/cureus.2861

**Published:** 2018-06-22

**Authors:** Mohsin Hamid, Ali R Ghani, Waqas Ullah, Usman Sarwar, Rajesh Patel

**Affiliations:** 1 Internal Medicine, Abington Jefferson Health, Abington, USA; 2 Pulmonary and Critical Care, Abington Jefferson Health, Abington, USA

**Keywords:** blow-hole incisions, diaphragmatic hernia

## Abstract

Spontaneous lung herniation is a rare phenomenon in which the lung parenchyma along with the pleural membranes protrudes outside their usual boundaries and can lead to a wide variety of complications. We are reporting a case of a middle-aged male who presented with chronic obstructive pulmonary disease (COPD) exacerbation with severe bouts of cough. Initial computed tomography (CT) chest was unrevealing, but two days later, he developed spontaneous lung herniation, which was initially managed conservatively, but later it progressed to pneumothorax, pneumomediastinum, with striking CT scan images showing extensive subcutaneous emphysema. Blowhole incisions were done on the anterior chest wall which led to ultimate recovery.

## Introduction

A hernia is an abnormal exit of tissue or an organ from the walls of the normal anatomic encasement in which it usually resides. A wide array of anatomic structures (e.g., intervertebral disc, abdominal viscera) can herniate, leading to different symptoms/manifestations, which may or may not require medical treatment(s). In the context of the lungs, herniation is a relatively uncommon occurrence that invariably requires medical treatment. Lung herniation is defined as the protrusion of the lungs beyond their usual confines through an abnormal opening in the chest wall. Lung herniation may be classified based on the cause of the lung herniation, which may be traumatic, iatrogenic, or spontaneous. Most of the reported medical literature regarding lung herniation pertains to traumatic or post-surgical lung herniation where physical manipulation/trauma gives rise to the condition. Roland first described the condition in 1499. Since the first description by Roland, the condition has remained remarkably rare with only a few published case reports [1-2]. Here, we are reporting a unique case of a small spontaneous lung herniation from coughing, which later got complicated by pneumothorax, severe subcutaneous emphysema, pneumomediastinum, and pneumopericardium.

## Case presentation

A 65-year-old man with severe chronic obstructive pulmonary disease (COPD) was admitted with a four-day history of chest pain and worsening shortness of breath. He explained the chest pain started suddenly when he tried to reach out for something on his computer table. It was located on the right anterior chest, sharp in nature, 7/10 in intensity, pleuritic, and worse with coughing and deep breathing. He had a past medical history of severe COPD with frequent exacerbations recently necessitating multiple antibiotics and steroid courses, coronary artery disease, gastroesophageal reflux disease (GERD), and hypertension. He had a 30-pack per year smoking history and quit about 10 years ago. He was a retired fireman living with his family.

**Figure 1:**
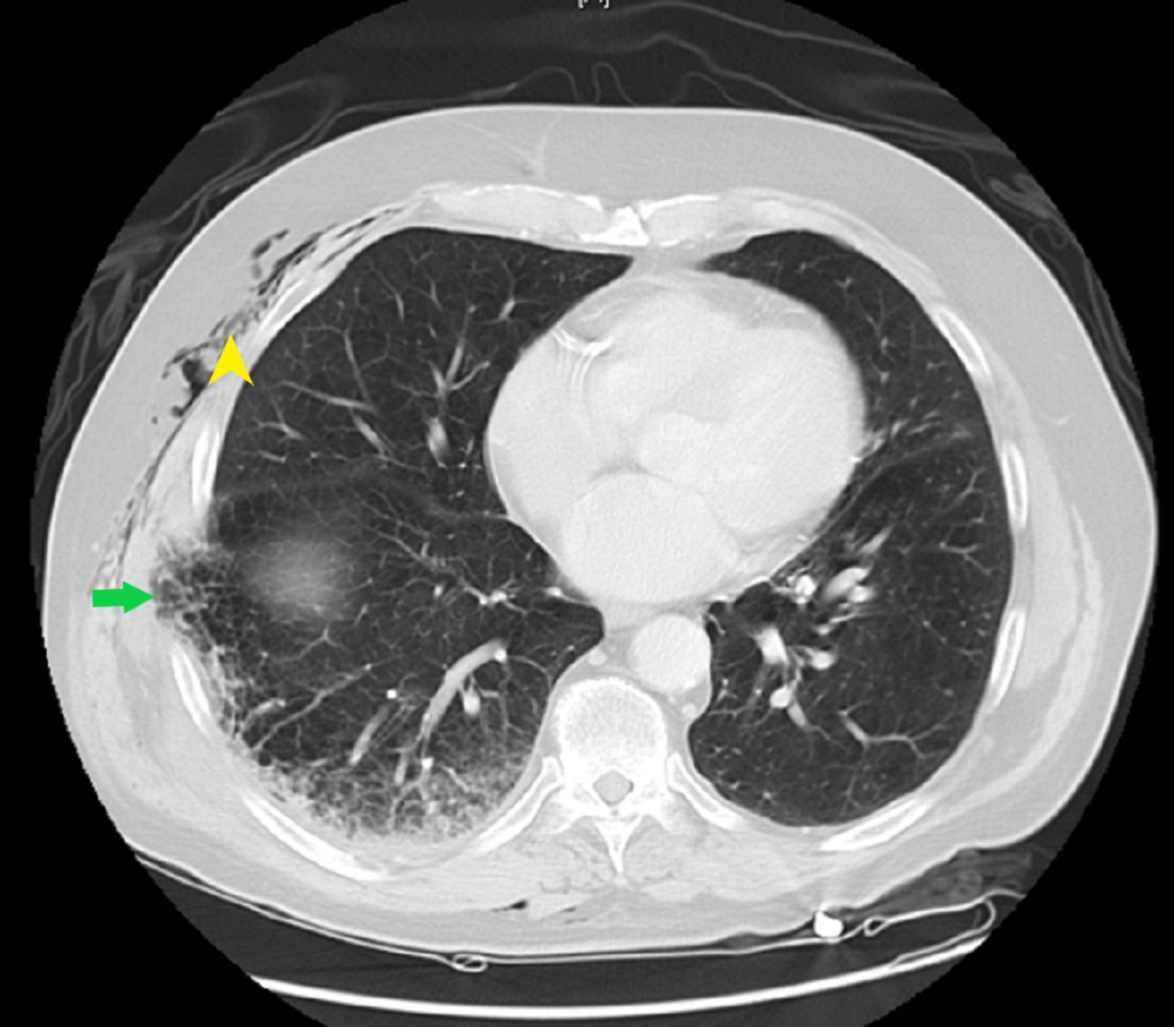
CT chest axial view showing a small portion of the right lung herniating through the right posterolateral seventh and eighth rib space (arrow) with mild subcutaneous emphysema (arrowhead) CT: computed tomography

Vitals signs in the emergency department (ED) were stable; he was breathing on ambient air. The physical examination demonstrated decreased breath sounds bilaterally without any wheezing or Ronchi. Moderate tenderness was present in the mid-axillary line in the fifth intercostal space, but no other abnormalities were noticed. Laboratory investigations were negative for any leukocytosis, troponin, or any other abnormalities. The electrocardiogram (EKG) showed a normal sinus rhythm. Computed tomography (CT) angiography was done to rule out pulmonary embolism (PE). The CT was negative for PE but showed mild peribronchial infiltrate in the right middle lobe and posterolateral lung herniation between the seventh and eighth ribs, with minimal subcutaneous emphysema along the right chest wall (Figure 1).

**Figure 2:**
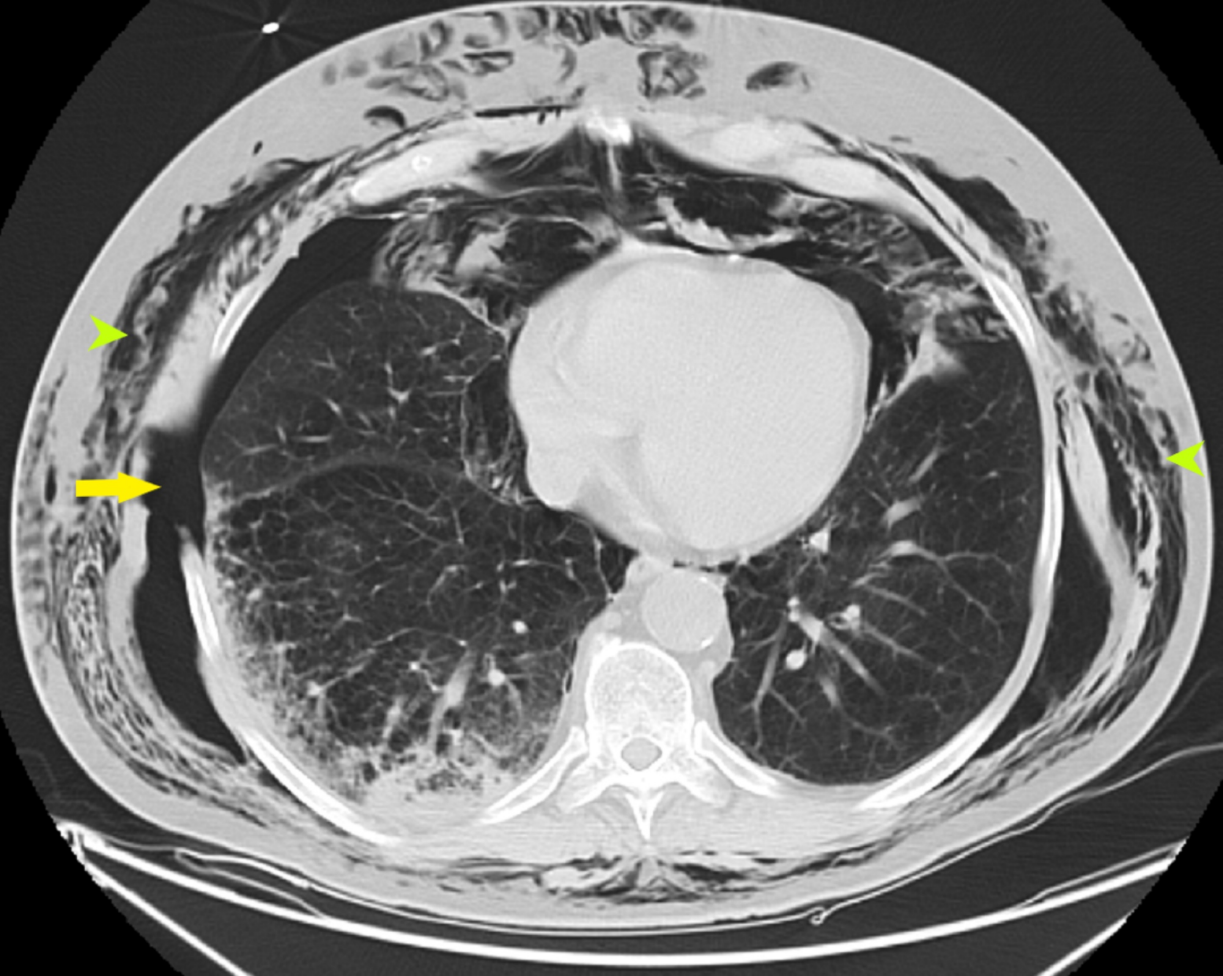
CT chest axial view showing large right-sided pneumothorax (yellow arrow) with extensive subcutaneous air (green arrowheads) throughout the thorax CT: computed tomography

**Figure 3:**
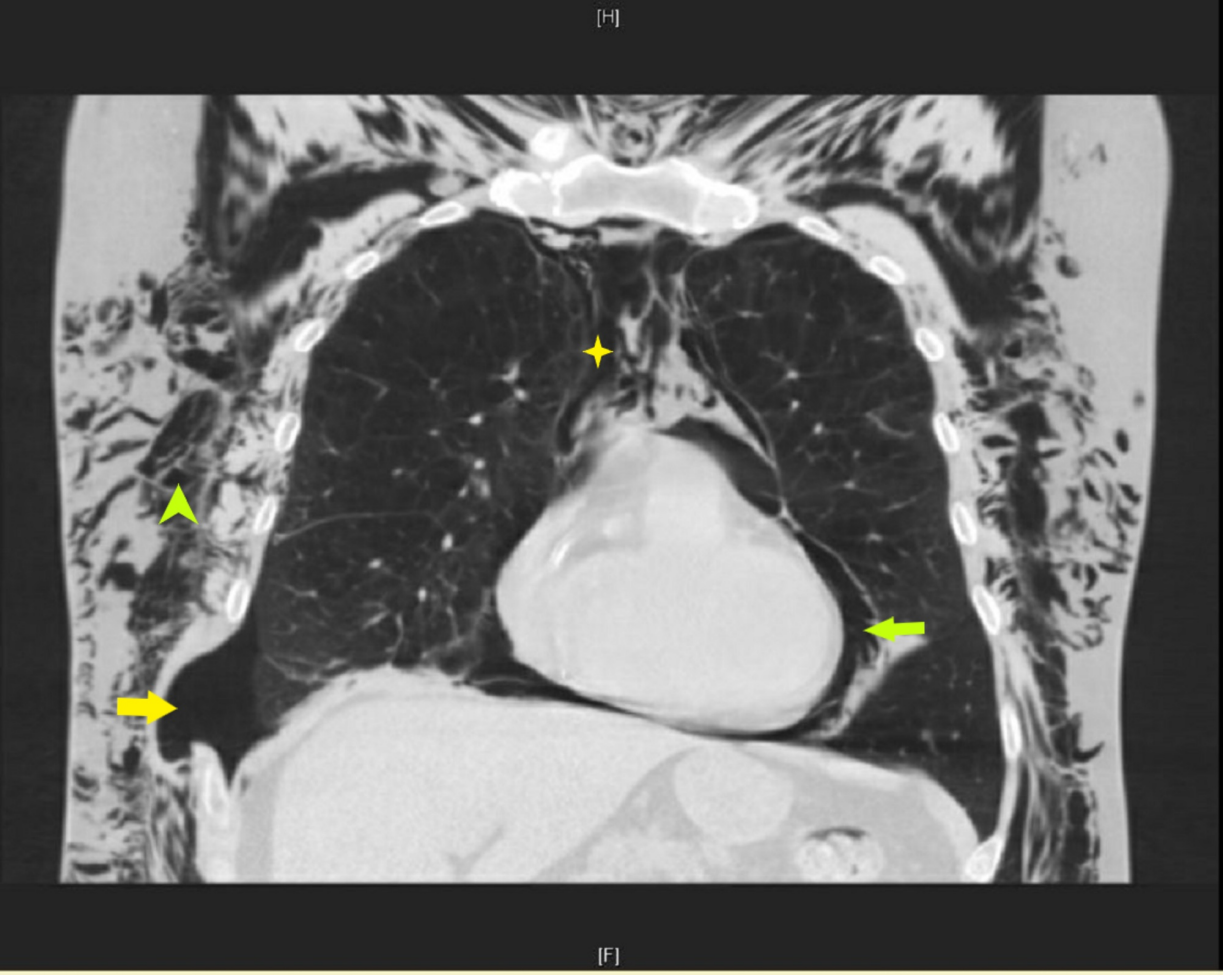
CT chest coronal section showing right-sided pneumothorax (yellow arrow) at the site of the previous herniation with extensive subcutaneous emphysema (green arrowhead), pneumomediastinum (yellow star), and pneumopericardium (green arrow) CT: computed tomography

The patient was admitted to the hospital and managed conservatively on broad-spectrum antibiotics, including vancomycin, levofloxacin, and 40 mg per day of prednisone. Two days later, his face swelled up suddenly with a change in the quality of his voice while he was eating dinner. An examination showed a swelling in the neck, diffuse crepitations on his body involving the face, all the way down to the buttocks. Repeat CT chest and neck showed extensive subcutaneous emphysema in the face, neck, chest, and mediastinum with a right-sided pneumothorax at the level of the previous lung herniation (Figures 2-3).

**Figure 4:**
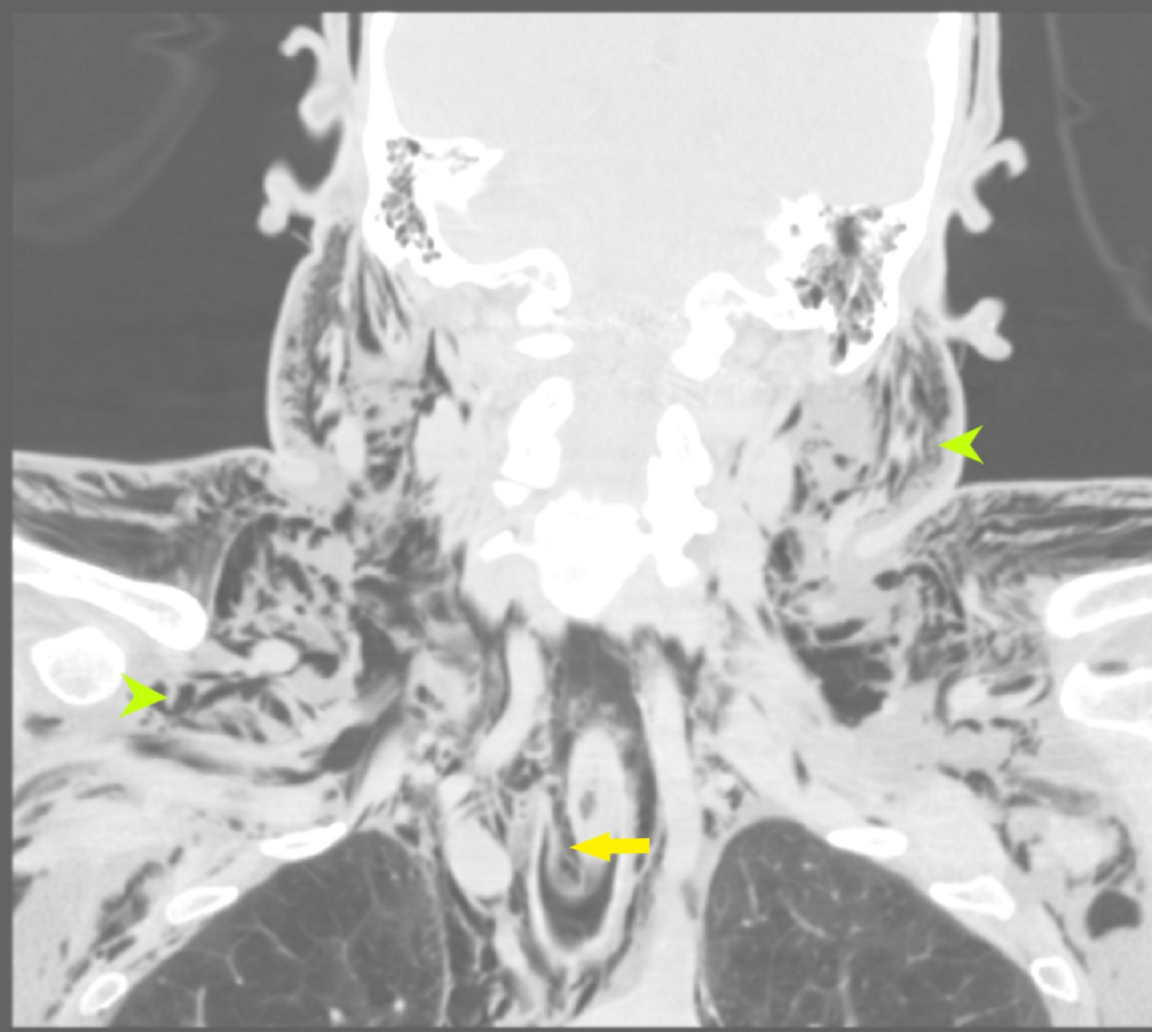
CT neck coronal section showing extensive subcutaneous emphysema (green arrowheads) involving the face, neck, and chest; narrowing of the oropharynx due to extensive air in the prevertebral space and retropharyngeal space is also present (yellow arrow) CT: computed tomography

The prevertebral and retropharyngeal air was demonstrated as compressing the oropharynx (Figure 4). His oxygen requirement went up to 6 liters nasal cannula. A blowhole incision was made on the anterior chest wall, and he was observed in the medical intensive care unit (MICU). Surgery to close the defect was deferred due to his other comorbidities and the higher risk of post-operative complications. He improved gradually over the course of the next few days, completed the course of antibiotics with steroids, and was discharged to a rehabilitation center and did well post-discharge.

## Discussion

Roland first reported lung herniation in 1499 and then in 1845, More-Lavalle classified lung hernias based on the location of protrusion and etiology [1,3] (Table 1).

**Table 1:** Classification of lung hernias

Classification according to location and prevalence
1. Cervical (one-third)
2. Thoracic (two-thirds)
3. Diaphragmatic (rare)

Classification according to etiology
A) Congenital
B) Acquired, can be subcategorized into:
i) Pathological (18%)
ii) Spontaneous (30%)
iii) Traumatic (52%)

The most common types of lung hernias are traumatic hernias acquired through motor vehicle accidents, war-time injuries, rib fractures, post-thoracic surgery, and chest compressions during cardiopulmonary resuscitation. Spontaneous lung hernias are rarely described in the literature, and most of the cases are associated with COPD, obesity, and smoking. The association of COPD and lung herniation is probably due to chronic hyperinflation and coughing, leading to raised intrathoracic pressure in the background of multiple courses of steroids that lead to muscular atrophy weakening the walls of the thoracic cavity [4].

The chest wall is heavily reinforced by muscles around it, which makes the herniation of lung tissue virtually impossible except in the anterior thoracic wall between the eight and ninth ribs, where the external intercostal muscles are lacking, and posteriorly from the coastal angle to the vertebrae due to the absence of intercostal muscles. These are the two most common places of spontaneous lung herniation [5].

The presentation of spontaneous lung herniation is usually a soft expansile swelling that worsens with cough or any other maneuvers that increase the intrathoracic pressure, such as coughing and sneezing. Mostly, spontaneous lung herniation is asymptomatic and is noticed on chest X-rays or CTs that are done for other reasons or on physical examination.

A chest X-ray can help diagnose the condition if the “lung beyond rib sign” or “lucent lung sign” is present, which means the presence of lung markings outside the bony thorax. However, X-rays have low sensitivity for ruling out herniation. A chest CT is virtually diagnostic in all cases, as it provides not only visual identification of any pulmonary parenchyma present outside the chest wall but also provides detailed anatomical information about the chest wall, including the bony structures and accompanying musculature. Splaying or widening of the intercostal space can also be seen at the site of herniation and may be mistaken for missed ribs by a radiologist without the appropriate history. The potential complications of lung herniation include lung incarceration, strangulation causing hemoptysis, lung necrosis, pneumothorax, and pneumomediastinum. It is important to note that smaller defects need to be closed earlier than the more substantial defects, as the risk of incarceration is higher [6].

**Figure 5:**
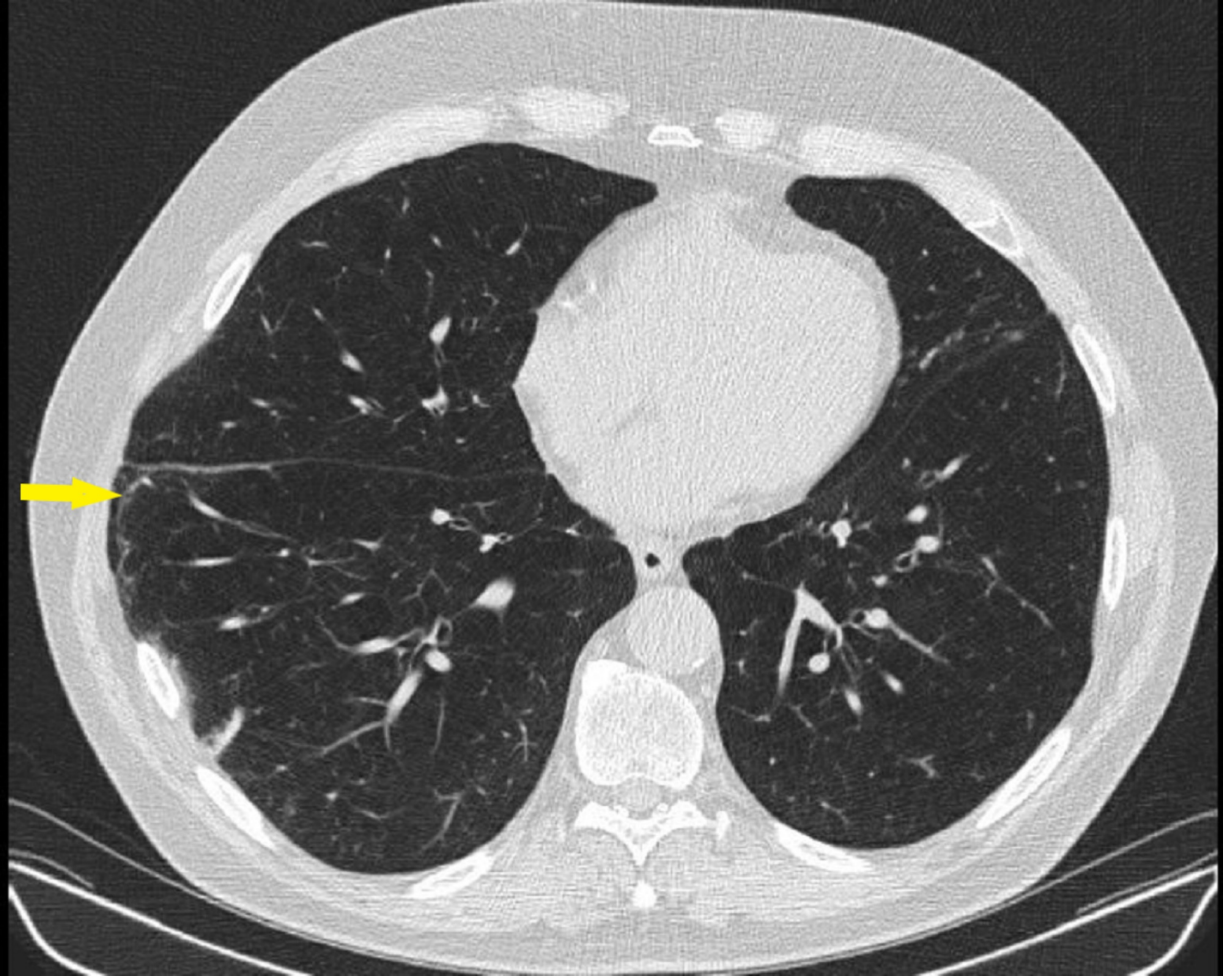
CT chest coronal view showing persistent lung herniation and splaying of ribs (yellow arrow) CT: computed tomography

There are no randomized controlled trials to determine the superiority of conservative versus surgical treatment and the treatment recommendations are based upon on case reports and small case series. Treatment decisions are made based upon the size, location, symptoms such as pain and dyspnea, and the patient’s comorbid conditions. Conservative management for asymptomatic herniation is done by anti-tussive medications, compressive pads, and corsets. However, if the pain is persistent or the size of a hernia is increasing, surgical intervention is recommended. Also, in anterior chest wall hernias, surgery is preferred over conservative management, irrespective of size, due to the potential extension of a lung hernia into a thoracoabdominal hernia. Surgical treatment can be done with thoracotomy or laparoscopically although for more substantial defects, reconstruction of the chest wall with muscle flaps or synthetic materials is mostly used [7]. The patient in our case was managed conservatively and recovered uneventfully. A CT chest scan done one year after the discharge showed persistent herniation but no other complications (Figure 5).

## Conclusions

Spontaneous lung herniation is a rare condition that can present with subtle symptoms, such as mild shortness of breath, acute chest pain, and localized ecchymosis. Cardiothoracic surgery should be involved early in the course for a discussion of operative versus conservative management even in the case of small hernias, as they are at high risk of strangulation.
